# A novel design process for selection of attributes for inclusion in discrete choice experiments: case study exploring variation in clinical decision-making about thrombolysis in the treatment of acute ischaemic stroke

**DOI:** 10.1186/s12913-018-3305-5

**Published:** 2018-06-22

**Authors:** Aoife De Brún, Darren Flynn, Laura Ternent, Christopher I. Price, Helen Rodgers, Gary A. Ford, Matthew Rudd, Emily Lancsar, Stephen Simpson, John Teah, Richard G. Thomson

**Affiliations:** 1 Institute of Health and Society, Newcastle University, Newcastle upon Tyne, UK; 2 School of Nursing, Midwifery and Health Systems, University College Dublin, Dublin, Ireland; 3 NIHR Newcastle Biomedical Research Centre based at Newcastle upon Tyne Hospitals NHS Trust and Institute of Neuroscience, Newcastle University, Newcastle upon Tyne, UK; 4 Stroke Research Group, Institute of Neuroscience, Newcastle University, Newcastle upon Tyne, UK; 5 Northumbria Healthcare NHS Foundation Trust, Ashington, UK; 6 Oxford University Hospitals NHS Foundation Trust, Oxford, UK; 7 Department of Health Services Research and Policy, Research School of Population Health, Australian National University, Canberra, Australia; 8 The Stroke Association, Gateshead, UK

**Keywords:** Discrete choice experiment, Intravenous thrombolysis, Clinical decision-making, Acute ischaemic stroke, Design process, Methodology, Patient vignettes

## Abstract

**Background:**

A discrete choice experiment (DCE) is a method used to elicit participants’ preferences and the relative importance of different attributes and levels within a decision-making process. DCEs have become popular in healthcare; however, approaches to identify the attributes/levels influencing a decision of interest and to selection methods for their inclusion in a DCE are under-reported. Our objectives were: to explore the development process used to select/present attributes/levels from the identified range that may be influential; to describe a systematic and rigorous development process for design of a DCE in the context of thrombolytic therapy for acute stroke; and, to discuss the advantages of our five-stage approach to enhance current guidance for developing DCEs.

**Methods:**

A five-stage DCE development process was undertaken. Methods employed included literature review, qualitative analysis of interview and ethnographic data, expert panel discussions, a quantitative structured prioritisation (ranking) exercise and pilot testing of the DCE using a ‘think aloud’ approach.

**Results:**

The five-stage process reported helped to reduce the list of 22 initial patient-related factors to a final set of nine variable factors and six fixed factors for inclusion in a testable DCE using a vignette model of presentation.

**Conclusions:**

In order for the data and conclusions generated by DCEs to be deemed valid, it is crucial that the methods of design and development are documented and reported. This paper has detailed a rigorous and systematic approach to DCE development which may be useful to researchers seeking to establish methods for reducing and prioritising attributes for inclusion in future DCEs.

**Electronic supplementary material:**

The online version of this article (10.1186/s12913-018-3305-5) contains supplementary material, which is available to authorized users.

## Background

A discrete choice experiment (DCE) is a method used to explore the relative importance of different attributes within a decision-making process. Based on random utility theory [1], DCEs operate on the tenet that there are multiple attributes influencing decision-making and that all decisions involve trade-offs between the range of elements that constitute the influential attributes. DCEs offer a means through which the nuances of decision making can be understood, by providing insights into the often implicit trade-offs made, which are not easily accessed through other research methods. DCEs have been increasingly adopted to examine attributes influencing decision-making in areas of healthcare, including stroke rehabilitation [2, 3].

Traditionally, the broad study design of a DCE is informed by literature review, expert opinion, theoretical arguments and/or qualitative work [4]. However, there is no standard development process for a DCE for identifying a comprehensive list of attributes that may influence the decision of interest and the subsequent optimal selection of attributes for inclusion in the final design, necessary due to the frequently large numbers and variable nature of type of attributes that may be identified. Furthermore, there is a lack of guidance on the optimal presentation of choice sets or decision alternatives in DCEs. The processes involved in the design of DCEs are often poorly described, with little or no detail reported regarding the procedures for selection of attributes/levels in the final design. In particular, there is a dearth of guidance on procedures that can be undertaken to select the most salient attributes and their associated levels from the full range of attributes that may influence the decision of interest and, when reported, this information has been characterised as “excessively brief” [4].

The International Society for Pharmacoeconomics and Outcomes Research (ISPOR) has published two reports on recommended best research practice in the development, administration, analysis and reporting of DCEs [5, 6]. However, they do not offer a clear approach to certain important aspects of DCE development; specifically how to select the most salient attributes/levels for inclusion in choice scenarios from the full range of attributes that may have an influence on the decision of interest; and administration in terms of the optimal method of presenting choice scenarios to respondents to maximise process/face validity of the choice task.

With some notable exceptions [7, 8], there is a dearth of examples of a clear, transparent DCE study design processes in the published literature. Although researchers have advocated the use of qualitative methods such as one-on-one interviews, focus groups, analysis of policy documents, and expert opinion to inform the design of DCEs [4, 9], recent evidence suggests that the uptake and reporting of qualitative methods to inform DCE design are lacking. Vass et al. [10] found that of 254 healthcare DCEs examined, 44% did not report using any qualitative methods and only 11% reported relying on qualitative methods extensively. This underuse, or lack of reporting of, qualitative methods to inform DCE development inhibits a clear understanding of whether a DCE design process has been rigorous and systematic and thus inhibits the ability to effectively evaluate the quality of the research. Quantitative approaches, including ranking exercises and nominal group techniques to support the process of selecting factors for inclusion in DCEs have been outlined [7, 8] but to our knowledge, there are no examples where both qualitative and quantitative methods have been employed to inform DCE design.

The validity of the results obtained from a DCE is contingent on the quality of the study design process [11]. When DCEs are conducted in circumstances where there is little or no empirical evidence to guide study design, documenting the design process is especially important. Current reporting on DCE design often does not provide sufficient information to allow for an informed judgement to be made regarding the quality of a study [4, 9]. Increased transparency at this stage of the process would offer valued guidance to researchers, allowing them to draw on approaches and methods that best suit the objectives at hand. It would also support more effective critical appraisal of the findings of the reported studies utilising DCEs. Robust methods should underpin DCE design, yet it is often not possible to judge these processes, or whether there was any structured development process involved, from the limited information presented in many published papers.

There have been calls for a minimum level of reporting on the DCE development process, including the processes used to collate an initial list of attributes, the analyses conducted during this design stage (including sample details and information on type of analysis conducted), processes undertaken in reducing attributes to a manageable number, and a brief description of the results of these processes [9, 10]. As each decision made during the DCE development process can have implications for both the final design and the validity of results obtained, the adherence of researchers to these basic reporting conventions would improve the transparency of DCE research and enable readers to better judge the quality of such studies. By employing a DCE to explore decision-making about thrombolysis in acute stroke care as an exemplar, we describe a systematic, transparent and iterative multi-method process to design an online DCE.

### Decision context: Thrombolytic treatment in acute stroke care

Intravenous thrombolysis using recombinant tissue plasminogen activator is an effective medical treatment for acute ischaemic stroke*.* Though there is clear evidence of its efficacy and benefit in certain patient groups and it is promoted and supported by national guidelines [12–15], it remains underused, as approximately one in five patients eligible for thrombolysis do not receive it [16]. Decision making about whether or not to treat a stroke patient with thrombolysis is complex due to the time limited window for treatment, potential difficulties in obtaining consent, and the many clinical attributes that might influence the balance between risk and benefit for individual patients [17]. Early treatment of acute ischaemic stroke with thrombolysis is associated with more favourable outcomes, yet there is a small but significant risk of adverse outcomes as result of treatment [18]. Symptomatic intracranial haemorrhage occurs as a complication in approximately 3–4% of patients, which increases the probability of long-term disability and death [16]. A DCE offers on optimal means of better understanding the complexities of clinician decision-making about intravenous thrombolysis in a way that reflects decision making in practice.

The aim of this paper was to build on existing DCE guidelines by describing a systematic and rigorous process for DCE construction and choice set presentation, using the exemplar case of clinical decision-making for thrombolytic treatment during acute stroke care.

## Methods

### Overview of DCE 5-stage design process

**Fig. 1:**
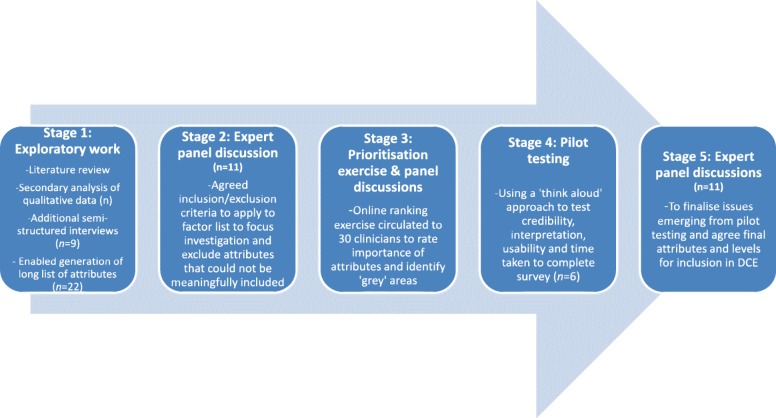
Summary of key stages of development process

We designed hypothetical patient vignettes to mimic the decision of interest as closely as possible, using a 5-stage iterative process described by Fig. 1. This was process was adopted because qualitative research was required to inform the design, but also in order to consider the nature and relative importance of factors influencing choice (clinical and non-clinical), and it was important to check the material being constructed and validity of the choices with clinicians. The decision of interest was in the form of a binary response (decision to offer thrombolysis or not) as this reflects routine clinical practice whereby clinicians are faced with one patient at a time and must decide whether or not to offer thrombolysis. Clinicians were likely to be familiar and comfortable with vignettes as they are regularly used in training and continuing professional development.

Ethical approval was obtained from Newcastle University Research Ethics Committee (reference: 00720/2013). Consent to participate was obtained for all those involved in the study.

### STAGE 1. Exploratory work

We considered all attributes that might be influential on thrombolysis decision-making, as omitting important attributes in the DCE may bias findings [2]. We drew on previously gathered qualitative data collected for a previous research programme examining decision-making in acute stroke [19] and on work to inform the development of a computerised decision support tool for thrombolysis in acute stroke care; COMPASS (*COMPuterised* decision Aid for *Stroke* thrombolysiS) [20]. This included qualitative interview data on the views and experiences of stroke clinicians about thrombolysis decision-making, ethnographic data collected through non-participant observation of thrombolysis decision-making discussions, and data from the usability and feasibility testing of COMPASS. Relevant literature was also reviewed to identify further attributes that have been documented to affect decision-making about thrombolysis and attributes influencing medical decision-making more broadly [21–26].

In addition, nine telephone-based semi-structured interviews were conducted with stroke clinicians and experts in stroke research. Purposive sampling was used to capture the views of experienced clinicians identified through the literature review (who had not contributed to the development of the COMPASS tool). We also targeted clinicians from hospitals in both the upper and lower quartiles of thrombolysis rates, based on figures from national audits [27]. These interviews aimed to identify any additional patient-related attributes or levels that represented the borderline and/or difficult cases; in particular the nature of trade-offs made between influential attributes and levels when making thrombolysis decisions. Telephone interviews were conducted due to the geographic spread of participants. Interviews were audio-recorded and transcribed verbatim.

Anonymised transcripts were imported into *QSR International’s* NVivo 9 to facilitate the coding and analysis of data using a framework approach, where the initial framework was guided by the interview guide (deductive approach) and additional codes were generated where required (inductive approach) [28]. Codes were themed where relevant and coding was discussed among the research team to reach consensus. Analysis of interview data and review of secondary data sources from the previous research programme on stroke [19], alongside relevant literature, facilitated the generation of an initial long list of potentially influential patient-related attributes on clinical decision-making regarding thrombolysis.

### STAGE 2. Expert panel discussions – Procedure for screening attributes and levels

Exploratory work commonly identifies too many attributes/levels to include in DCEs and, due to sample size constraints and the potential for respondent cognitive burden, it is often not practical or feasible to include all possible combinations that may influence decision-making. By including all potentially influential attributes and levels of interest, the statistical power of a DCE to detect effects may be reduced, due to the large number of possible combinations of levels and the inability of a small sample size to adequately assess all these various combinations. Methods are needed to select the most important attributes and levels. Although there are publications that provide generic advice on DCE design [4, 9], there is a dearth of explicit guidance on this process. A well-designed DCE has been described as “one that has sufficiently rich set of attributes and choice contexts, together with enough variation in the factor levels necessary to produce meaningful behavioural responses.” [29] Abiiro et al. [7] advise employing quantitative methods, such as ranking exercises, to support the process of selecting attributes for inclusion to a manageable number. Prior to the ranking exercise at the next phase, the expert multidisciplinary panel in the current study (consisting of three stroke clinical researchers, one trainee stroke physician, two patient representatives (from the UK Stroke Association), two chartered psychologists, two health economists, and an expert in shared decision-making) screened the long list of attributes (generated during Stage 1) in terms of whether they would be feasible or meaningful to include in a DCE, which would then be further scrutinised using a structured prioritisation/ranking exercise. Following this ranking exercise and subsequent discussions, a set of inclusion and exclusion criteria were agreed with reference to the study objectives.

### STAGE 3 - phase 1. Design of online structured prioritisation exercise (SPE)

In order to identify the relative importance of various patient-related attributes for clinical decision-making and areas of uncertainty on specific attributes, an online structured prioritisation exercise (SPE) was developed. This was a ranking exercise performed by clinicians on a single occasion to prioritise attributes related to clinical decision-making for inclusion in the cases, to ensure that the DCE was relevant without being excessively long. It offered a means to elucidate a broader range of views to inform the selection of the attributes and in particular, questions were phrased to identify where uncertainty and ‘cut-offs’ existed on individual attributes regarding the suitability of the patient for thrombolysis. This step was important for the current study as it was widely recognised that individual clinicians had different thresholds or cut-offs for decision-making. The findings would inform expert panel discussions in the next phase of the design process. Free text boxes were also provided after each question. The SPE was hosted on *Qualtrics* (www.qualtrics.com) and 30 stroke clinicians in the north east of England were invited to participate. The full SPE is available in Additional file 1.

### STAGE 3 - phase 2. Using the SPE to inform selection of attributes/levels

The same expert panel assembled in Stage 2 then independently ranked each attribute in order of priority for inclusion in the DCE, indicated how they would operationalise them, and provided suggestions on possible levels for each patient-related attribute. Results were then aggregated and fed back to the panel to inform discussion on selection of the final list of attributes and levels for inclusion. There was a need to compromise on the perceived importance of attributes to ensure concerns regarding sample size/design feasibility, ecological validity (i.e., ensuring information presented is as consistent as possible with information that would be available in a real life situation) and the potential impact of unconscious attributes (e.g., patient ethnicity) were considered in the final attribute selection process. Issues considered during these panel discussions were:

#### Ensuring clinical face validity

Realism of the patient information presented in vignettes and that attributes/levels could plausibly occur together was vital. Certain attribute levels could not reasonably appear together meaningfully and therefore some initial constraints were imposed on the design. For instance, a modified Rankin score of 0 or 1 (indicating patient is able to carry out all usual duties and activities) is implausible for a patient described as having severe dementia.

#### Ensuring sufficient information was present in vignettes

It was crucial that information on certain attributes was provided in the vignettes (as either variable or fixed attributes) in order for clinicians to reach a decision about the offer of thrombolysis and for the decision-making process to mimic real-life decisions as closely as possible. For instance, stroke severity score and the results of the CT scan were considered as vital information.

#### Anticipated sample size and resulting design considerations

Whilst more levels can define the true relationship between attributes and their influence on decision-making, there is an inherent trade-off between this and the number of attributes and levels that can feasibly be included in the DCE design [1]. Furthermore, there are important considerations regarding the maximum numbers of attributes and levels a participant can process simultaneously. Due to the limited size of the pool of potential study participants in the current research, restricting the number of variable attributes and levels per factor was necessary to control the number of potential combinations in order to ensure the DCE would have sufficient statistical power to detect important effects. Based on previous studies and estimates of the total number of clinicians treating acute stroke patients in the United Kingdom, a sample size of 150–200 was estimated as achievable in the current study. This estimate represents approximately 25–45% of the 422 clinicians registered as ‘Full Members’ of the British Association of Stroke Physicians [30]. Moreover, to address cognitive burden, we judged that 12–16 hypothetical patient vignettes would be the upper limit of decision vignettes that should be administered to each participant (and this would be tested/clarified in the piloting of the DCE in Stage 4).

Attributes that were considered important to provide basic clinical information (but not included as variables in the DCE) were included as fixed attributes in the design to remove subjectivity around interpretation of additional issues. This provides common interpretation of such attributes across respondents, whilst retaining face validity. In the current study, blood glucose level was included as a fixed attribute in each vignette (6.0 mmol/L). An identical text description of a patient’s CT scan result was included in all vignettes to remove subjectivity around imaging interpretation. Definitions were also included to standardise the interpretation of attributes and levels. For instance, the standard definitions for modified Rankin scores [31] used to assess pre-stroke dependency were presented in each vignette. Definitions were also provided for pre-stroke cognitive functioning and the calculation for each level of stroke severity using the National Institute of Health Stroke Scale (NIHSS) [32] to ensure consistent interpretation.

#### Consideration of explicit versus implicit influences on decision-making

There is strong evidence that implicit attributes can be influential in medical decision-making [23]. Therefore the expert panel also considered attributes that did not emerge from the exploratory work and SPE. For instance, gender may have a population-level effect on thrombolysis administration, with evidence of under-utilisation in women [33], although this may reflect a different age and presentation profile in women [34]. This is despite a pooled analysis of randomised controlled trials demonstrating that women are more likely than men to benefit from thrombolysis [35, 36]. Gender was not considered to be a conscious consideration at the individual, bed-side level by the panel. Other attributes that may have an implicit effect on decision-making were ethnicity and social class. Panel discussions were focused on striking a balance between the inclusion of both implicit and explicit attributes, which may influence thrombolysis decision-making.

### STAGE 4. Pilot testing

A pilot DCE was developed based on the decision made by the expert panel. The generation of a fully balanced design with all possible combinations of attributes and levels was not possible. Therefore, the software programme, *NGene* (v1.1.1; http://www.choice-metrics.com/), was used to generate a fractional factorial (d-efficient) design to generate a design that was as balanced as possible, given the imposed constraints. A fractional factorial design was employed due to the relatively large number of variable attributes (nine) included in the final DCE, with number of levels varying from two to six on different attributes. This allowed for the testing of a subset of possible combinations. Use of a blocked design allowed the number of vignettes presented to any one participant to be reduced to a number considered manageable in pilot testing to avoid overburdening participants. In each block, 13 hypothetical patient vignettes were presented to each participant.

The pilot testing of the initial DCE employed a ‘think aloud’ approach that was guided by best practice guidelines [37]. This method asks participants to verbalise what they are thinking when responding to the survey, which can help to reveal vague or confusing questions, or other issues in need of clarification. The aim of this pilot stage was to facilitate further testing of the credibility of the vignettes among the population of interest, examine participants’ understanding and interpretation of the task and questions, check the usability of the survey on different systems and browsers, and gauge how long the survey might take to complete. Changes were made to the design and wording of vignettes and phrasing of questions during the piloting process to ensure any amendments could be tested. Furthermore, post-pilot testing was undertaken by the expert panel prior to the formal data collection phase. The pilot testing protocol is available in Additional file 2.

### STAGE 5. Final expert panel discussions

At this stage, the same expert panel from Stage 2 were engaged to finalise the DCE. Refinements were made to the online survey based on outcomes of pilot testing and final amendments to questions and the instructions to the DCE were agreed.

## Results

Given the underreporting/lack of development work in DCE design in the field, we have developed a 5-stage process which offers a replicable and transparent strategy, with methods to identify a comprehensive list of attributes that may potentially influence a decision of interest. Underreporting of the design process highlights that procedures used to select the most salient attributes for inclusion in the final design need to be based on explicit criteria to permit a judgement about bias and external validity of the findings to be made. Furthermore, presentation of choice sets in DCEs using traditional methods may lack sensitivity to decision-making contexts, which indicated a need to identify the optimal mode of presenting choice sets to respondents to maximise face/clinical validity of the choice task that is important for augmenting external validity of findings from DCEs.

### Stage 1. Full list of attributes identified from initial exploratory work

Table 1 displays all the patient-related attributes identified as potentially important in thrombolysis decision-making based on: (i) the analysis of new and existing qualitative data sets on the views and experiences of stroke clinicians involved in thrombolysis decision-making; and (ii) a review of relevant literature.

**Table 1:** Patient-related attributes that could influence decision-making about thrombolysis

1.	Systolic blood pressure
2.	Diastolic blood pressure
3.	Blood glucose level
4.	Patient frailty
5.	Stroke severity (NIHSS score)
6.	History of hypertension
7.	History of stroke
8.	Anticoagulation status / INR level
9.	Aspirin monotherapy
10.	A patient’s level of social support
11	Major surgery in past 3 months
12.	Presence of diabetes at time of presentation
13.	Patient age
14.	Patient ethnicity
15.	Patient gender
16.	Socioeconomic status of patient
17.	Stroke onset time to treatment
18.	Recent infarction on CT/MRI scan
19.	Pre-stroke cognitive functioning / capacity / comprehension of risk information
20.	Pre-stroke dependency status
21.	Patient/relative values, knowledge and views on thrombolysis
22.	Co-morbidities

### Stage 2. Expert panel discussions

The expert panel reviewed the list generated by Stage 1 and concluded it was comprehensive and relevant. There was a need at this stage to begin the process of reducing the list to the most important attributes. A set of criteria were agreed to focus the scope of the study:

#### Removal of attributes that could be considered as related to uncertainty regarding the diagnosis of acute ischaemic stroke

This was considered prudent based on the rationale that: (i) incorporating diagnostic uncertainty has methodological implications for the design of the patient vignettes and interpretation of results from the DCE. Given that a diagnosis of acute ischaemic stroke represents a gradient of certainty (and includes consideration of differential diagnoses such as transient ischaemic attack and stroke mimics) this does not lend itself well to the DCE framework. Furthermore, diagnostic uncertainty was beyond the scope of the aims stated in the study protocol [38] to explore attributes influencing decisions to offer thrombolysis, as opposed to what attributes influence diagnosis.

#### Removal of redundant/uninformative questions from the SPE

Questions were excluded if the panel agreed that the area of uncertainty on any attribute had been clearly identified by the literature and/or qualitative data. For instance, a question regarding patient’s pre-stroke status using the modified Rankin Scale (mRS) was removed as the panel were confident that most clinical variation in decision-making exists between mRS 2 and 3.

Furthermore, attributes were omitted that have been shown to have a population level effect in research studies but were not considered by the expert panel (or were not identified in exploratory interviews) to be important for decision-making about thrombolysis at the individual patient level (e.g., current use of aspirin).

### Stage 3 (phase 1). Results of on-line survey (SPE)

The online SPE was live for six weeks and 11 clinicians completed the survey (37% response rate). Results are summarised in Additional file 3.

### Stage 3 (phase 2). Results of second round of expert panel discussions

The ISPOR report on good research practices advises that discussion with experts is an approach that can be employed to help reduce the list of attributes [6]. The expert panel completed a ranking exercise to generate discussion and to help work towards a consensus regarding the importance of attributes for inclusion, and which attribute levels should be varied and which should be fixed. The results of this exercise are presented in Table 2. In consideration of the potential influence of implicit attributes on decision-making about thrombolysis, the panel elected to include ethnicity and gender as variable attributes in the DCE. Socio-economic status was also considered for inclusion in the DCE. However, due to the challenging nature of representing this information in vignettes and the potential influence of type of employment (e.g. manual work) in judgements of risk/benefit, it was decided to omit this attribute. Based on these discussions, it was agreed to include seven fixed attributes and nine variable attributes (see Table 3).

**Table 2:** Stage 3 (Phase 2) Expert panel ranking exercise results (*n* = 6)

Attribute	Mean rank (SD)^a^	Median	Suggested levels
Systolic BP	1.67 (1.2)	1	• 140 mm/Hg
			• 175 mm/Hg
			• 180 mm/Hg
			• 185 mm/Hg
			• 200 mm/Hg
Pre-stroke dependency status	4 (2.6)	4	• mRS 1
			• mRS 2
			• mRS 3
			• mRS 4
Pre-stroke cognitive status	5.33 (2.6)	5.5	• No impairment
			• Mild impairment
			• Moderate impairment
			• Severe impairment
Stroke Severity (NIHSS)	6.5 (2.9)	7	• NIHSS 2
			• NIHSS 3
			• NIHSS 5
			• NIHSS 23
			• NIHSS 25/26/27
SBP (after reducing)	6.6 (4.7)	5	• 185 mm/Hg
			• 190 mm/Hg
			• 200 mm/Hg
INR/Anticoagulation	6.7 (2.7)	5.5	• < 1.6
			• < 1.7
			• < 1.8
			• 2
Diastolic BP	6.8 (5.2)	6	• 100 mm/Hg
			• 110 mm/Hg
			• 115 mm/Hg
			• 120 mm/Hg
Frailty	6.8 (3.9)	7	• Composite measure using comorbidities, description of needs (i.e., walking stick)
			• “patient you [do not] perceive as frail”
Time since symptom onset	7.8 (6.1)	7	• < 1 h
			• < 3 h
			• 4 h
			• 4 h 15/20 mins
Recent major surgery	8.3 (3.6)	7.5	• Percutaneous coronary intervention
			• Hip replacement
			• Laparotomy
Previous stroke	9.2 (4.3)	10.5	• Combine with stroke severity?
			• 2 weeks ago
			• 4 weeks ago
			• 3 months ago
Comorbidities	9.8 (2.6)	10	• Disability-related
			• Chronic disease
			• Illness presenting bleeding risk
Blood glucose level (mmol/L)	10.8 (3.1)	11	• 16/19
			• 22
			• 25
			• 27
Patient/relative preferences	11.4 (2.6)	12	• No family present
			• Family present and eager for patient to be treated
			• Family present and worried about bleeding risk
Willingness to treat blood glucose	11.6 (3.7)	11	• Option to treat before thrombolysis decision
			• Option to present already treated level
Patient age	12.7 (5.7)	14.5	• 62/68/75
			• 80 / 8585
			• 95
Social support	15 (3.1)	16	• Indicator of dependency?
			• Use as part of frailty composite measure?
Presence of diabetes	15.4 (2.4)	16	• No history of diabetes
			• Patient has diabetes

^a^* Note*: Lower mean rank indicates higher perceived importance

**Table 3:** Fixed attributes used in DCE and rationale for inclusion

Attribute	Rationale for inclusion	Fixed level	Rationale for level
Blood glucose level	Variable levels may result in diagnostic uncertainty	6 mmol/L	Average blood glucose level based on SITS data of treated patients
CT scan text description	To avoid skill/subjectivity around interpretation of scans	CT scan was conducted and is consistent with ischaemic stroke; it shows no haemorrhage or new ischaemic changes	Decided not to include image due to potential variability in CT image interpretation skill and subjectivity; difficulty finding scans to match multitude of various patient characteristics. Text description deemed most appropriate to remove diagnostic uncertainty
	To ensure confirmation of diagnosis of acute ischaemic stroke		
Anticoagulation status	While it was deemed an influential attribute, only minority of stroke patients take an anticoagulant and therefore it was not included as variable attribute	patient is not on anticoagulation therapy	To avoid any issues surrounding INR levels that could complicate the decision to offer thrombolysis
Bleeding risk / recent surgery	Only relevant for a minority of patients. Challenging to operationalise variable and comparable levels in vignettes	no recent history of major bleeding	
Diabetes	Not ranked as important in vignettes	no history of diabetes	
	Included as fixed attribute for clinical validity		
Patient consent/ family assent		assume either patient consent of family assent is available for treatment	
Other / Comorbidities		there are no other attributes which would deter treatment	Due to difficulty defining fully and generating comparable and feasible levels of comorbidities. Potential overlap with pre-stroke cognitive and pre-stroke dependency status
Fixed attributes included post-pilot testing (Stage 4)			
Handedness of patient		“All patients are right-handed”	To clarify and ensure the deficits will be interpreted consistently across all level of stroke severity (NIHSS)
Licenced dose bolus preparation time		“can be prepared for administration within 5 min”	Pilot testing revealed that participants would attribute in variable times in their decision-making so stating this will help control this potential error

The expert panel also considered the attribute levels for inclusion and discussions were informed and guided by the results of the exploratory work and by the SPE, which specially explored the ‘grey’ areas of decision making, or attributes where respondents felt there was inadequate evidence to inform decision-making (e.g., cognitive impairment). Based on the staged process, the expert panel reviewed results and selected attribute levels to explore these ‘grey’ areas of decision-making.

### Stage 4. Results of pilot testing

Sixteen clinicians were invited to take part in pilot testing. Six agreed to take part and fully completed the pilot. The ‘think aloud’ approach helped to refine language used in the survey, ensured questions were interpreted by participants as intended, and confirmed sufficient information was presented in the vignettes to allow clinicians to reach a decision. The testing revealed that vignettes were credible, although a number of implausible combinations were identified and added as constraints to the design. These included low NIHSS scores with aphasia combined with moderate/severe dementia. These combinations were therefore excluded from the design.

Unexpected issues also emerged. For example, when two clinicians read that symptom onset began 4 h and 15 min ago, they factored in time to prepare the thrombolysis bolus into their decision-making (as the thrombolysis time window for treatment is up to 4.5 h post symptom onset). Therefore, thrombolysis bolus preparation time was included as an additional fixed attribute, stating that the treatment dose could be prepared within 5 min to eliminate this as an influence on clinicians’ decision-making (Table 3).

### Stage 5. Final attributes and levels for inclusion in DCE

Final amendments were made by the expert panel based on feedback received during the pilot stage. As pilot participants felt that the patient vignettes contained sufficient information to allow them to reach an informed decision about the offer of thrombolysis, the nine attributes (and associated levels) used in the pilot were retained for inclusion in the final DCE (Table 4). Definitions for variable attribute levels in the vignettes were also finalised by the expert panel based on the feedback from pilot participants (see Additional file 4).

**Table 4:** Final list of variable attributes and levels in the DCE

Attribute	Levels	Rationale
1. Systolic blood pressure	a. 140 mm/Hg^a^ b. 185 mm/Hg c. 200 mm/Hg	• Highest ranked attribute in Stage 3 phases 1 and 2 • Levels include those across rage from ‘safe to offer thrombolysis’ to ‘grey are’ to ‘outside the thrombolysis licencing guidelines’
2. Gender	a. Male^a^ b. Female	• To increase clinical face validity • To examine if gender has an unconscious influence on decision-making
3. Age	a. 68^a^ b. 85 c. 95	• Included for purposes of ecological and face validity • Evidence from exploratory work that some clinicians may take patient age into account and adhere to current licensing guidelines
4. Frailty	a. you do not perceive as frail^a^ b. you perceive as frail	• Very challenging to adequately define frailty due to subjectivity in how clinicians view/consider it • Aim was to trigger perception of frailty in patient and therefore the current phrasing was considered optimal to meet aim
5. Time since symptom onset	a. 50 min^a^ b. 2 h 30 mins c. 4 h 15 mins	• Potential greater benefit of very early treatment time (50 mins) included to compare to mid-point in time window and rapidly approaching end of window (4 h 15mins)
6. Pre-stroke dependency (mRS)	a. mRS 1 b. mRS 3 c. mRS 4^a^	• Qualitative work suggested mRS 3 was the grey area in dependency
7. Pre-stroke cognitive functioning	a. No history of memory problems^a^ b. Moderate dementia c. Severe dementia	• Exploratory work suggested dementia/cognitive functioning could influence decision to offer thrombolysis
8. Ethnicity	a. white^a^ b. Afro-Caribbean c. Asian	• Included as an attribute that may have an implicit effect on decision-making • Included as this is information that would be obvious in a typical decision
9. NIHSS (stroke severity)	a. NIHSS 2 (without aphasia)^a^ b. NIHSS 2 (with aphasia) c. NIHSS 5 (without aphasia) d. NIHSS 5 (with aphasia) e. NIHSS 14^a^ f. NIHSS 23	• Presence or absence of aphasia deemed very important in previous stage and therefore was included at lower NIHSS scores (mild strokes) to assess whether it would influence decision-making. • NIHSS 14 included as it is the SITS mean score (for treated patients) • NIHSS 23 considered a severe stroke

^a^reference category

### Key considerations and final design of DCE

**Fig. 2:**
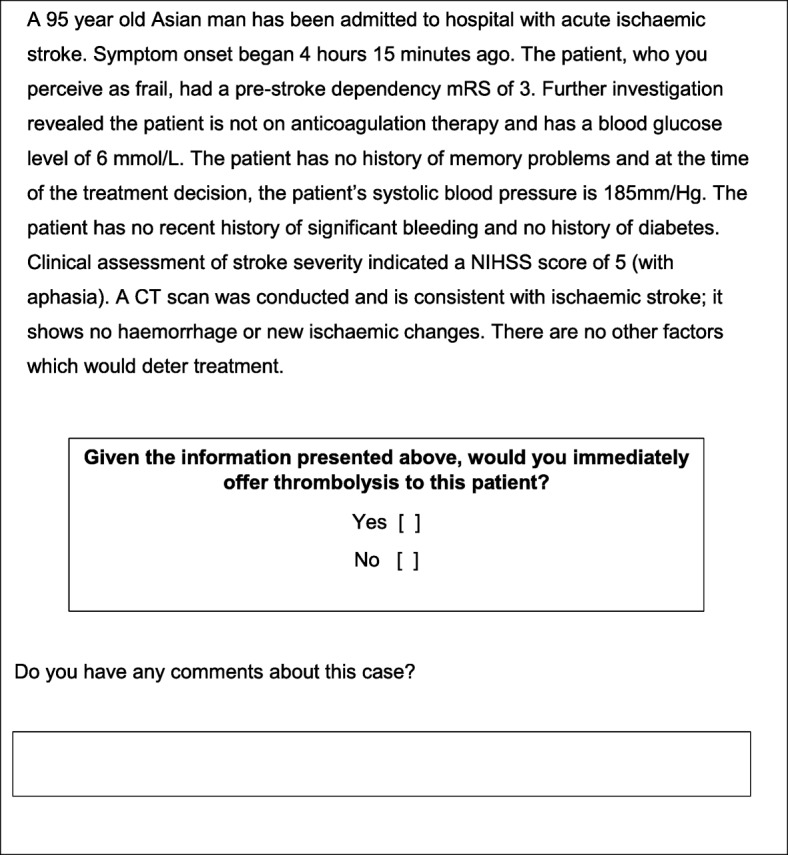
Sample patient vignette

Key considerations of the development of this online DCE included the size of the population of interest, the number of combinations of attribute levels, respondent burden and likely response rate. It was crucial for the expert panel to consider these issues alongside the perceived importance of various attributes and levels. Based on this five-stage design process, we were confident that we had provided sufficient information to allow clinicians to reach a decision by using both fixed and variable attributes. A sample vignette is displayed in Fig. 2 to demonstrate how the hypothetical patient attributes and levels were presented to clinicians.

## Discussion

The aim of this paper was to build on existing DCE design guidelines by describing a systematic and rigorous development process using an exemplar case describing the development of a clinically-valid DCE to investigate clinical decision-making about thrombolytic treatment in acute stroke care.

Previous literature has often pointed to the underreporting of aspects of DCE design. The under-reporting (or lack of DCE development work) on crucial aspects of this design process makes a strong case for adding additional details to the minimum reporting guidelines in order for readers to judge quality and to lend confidence in DCE results. Despite existing guidance on DCE design, development and evaluation [2, 4] and calls to improve reporting on this process [4], underreporting is still prevalent [10]. However, as noted by others [11], this may be due to tensions between journal editors and authors regarding space constraints and word limits. Nonetheless, journals are increasingly offering authors the opportunity to include supplementary material as online appendices and we urge authors to avail themselves of this, where possible, to ensure adequate and comprehensive reporting on DCE research.

The value and strength of the results obtained from a DCE are contingent on the quality of the study design process. When DCEs are conducted in circumstances where there is little or no empirical evidence to guide study design, documenting the design process is especially important. Increased transparency at this stage would offer guidance to researchers, allowing them to draw on approaches and methods that best suit the objectives at hand, or to develop, extend or replicate existing DCEs. Robust methods should underlie DCE design, yet it is often not possible to judge these processes, or whether there was in fact any development process involved, from the limited information presented in many published papers.

Previous work by Coast et al. [4] indicates that underreporting of processes to design DCEs is a problem in the wider DCE published literature. As each decision made during the DCE development process can have implications for the final design and the results obtained, we strongly urge that, at a minimum, researchers should be required to report details of the development processes of the DCE design including the rationale for the approach adopted, details of analyses conducted, processes to narrow down attributes/levels, results of analyses, and details of the sample. Furthermore, piloting of the DCE is a crucial step and researchers should document what the aim of piloting was, the results of piloting, and provide details on the sample used and any changes made post-piloting. Finally, the content and face validity of the DCE must be considered and maximised during the design process in order for the study and its results to be considered meaningful. The adherence of researchers to these basic reporting conventions would improve the transparency of DCE research.

To exemplify this, we have established and described an iterative process that is transparent, rigorous and systematic, providing rationales for decisions made at each stage of the DCE design. This five-stage development process was used to design an online DCE utilising choice sets in the form of patient vignettes to examine attributes that influence the clinical decision to offer thrombolysis to acute ischaemic stroke patients. A review of relevant literature and secondary analysis of qualitative data in Stage 1 enabled the compilation of a comprehensive list of attributes that might influence clinical decision-making about thrombolysis. Through expert panel discussions, and applying explicit inclusion/exclusion criteria, Stage 2 enabled selection of attributes/levels that could be meaningfully included in the choice sets of this DCE. Discussions in Stage 2 informed the design of a structured prioritisation exercise (SPE) in Stage 3 that consisted of a ranking exercise to prioritise attributes and levels for inclusion in the patient vignettes. Findings of ranking exercises guided subsequent expert panel discussions to reach consensus on the DCE choice sets and on-line survey design. This was followed by a pilot phase incorporating a think aloud approach (Stage 4), and in turn finalising the design of the DCE choice sets using fractional factorial methods (Stage 5).

This paper contributes to the literature on DCE development by clearly documenting a systematic and rigorous, iterative process to derive the final number and type of attributes, attribute levels and associated operational definitions for inclusion in an online DCE. It builds on ISPOR guidelines [5, 6] by providing explicit guidance on a five-stage process, which may be particularly beneficial when there is little or no previous research to guide DCE design. This transparency in the development process of a DCE (process validity) to understand the decision of interest (decision to offer thrombolysis or not), as well as information used by clinicians in their decision-making, confers confidence in the rigour and reliability of findings and applicability to the real-world (ecological validity) [39]. Although we have outlined a linear approach to achieve this, this development process is flexible and may adapted as appropriate.

This development work highlights the crucial importance of an iterative design process for augmenting the clinical face validity of the choice task in terms of (i) mode of administration (paper-based or electronic); (ii) identification and optimal selection of variable and fixed attributes and associated levels to include in choice sets; (iii) form of choice set presentation (textual versus traditional tabular approach, and order of information presentation;) and (iv) the necessity of piloting to augment clinical face validity, and in turn external validity, and test acceptability of choice sets by the target population, prior to the data collection phase.

Identification and optimal selection of variable and fixed attributes, and associated levels, to include in choice sets is critical. Given the limited size of the population of interest in the current DCE, the size of the research sample (based on explicit inclusion criteria) and likely response rate were important considerations to establish the feasible DCE design options that were likely to be achievable and testable within an acceptable level of statistical power. Additional issues to consider alongside selection processes are (i) whether levels of attributes cohere together meaningfully within choice sets to identify any potential design constraints, (ii) whether the information within choice sets provide sufficient information to clinicians to make a decision (i.e., are specific levels of fixed attributes warranted for the purposes of clinical face validity), and (iii) if the levels of variable attributes elicit variability in the respondents in terms of the clinical decision-making about thrombolysis.

The presentation of attributes and levels in the form of hypothetical patient vignettes in accordance with preferences of clinicians differs from the traditional form of presentation of DCE choice sets and constituted a novel aspect of the study. The intended respondents are familiar with this form of information presentation during clinical training; this was therefore the optimal method of information presentation to enhance clinical and external validity. Furthermore, attribute order/placement in the vignette was decided based upon the information that would be available to the clinician immediately and the typical order in which additional information may be obtained. The order of presentation of attributes was also informed by work to develop the COMPASS decision support tool [20].

Whilst this DCE was designed for the specific purpose of elucidating attributes influencing the decision to offer thrombolytic therapy for patients with acute stroke, we contend that a key strength of this study is the development of a five-step approach that can be transferable to other research contexts. We believe the steps undertaken can assist researchers in determining the attributes of interest and ensuring broad input into the DCE design process. The number of interviews to conduct with expert informants or the number of participants required to adequately inform an attribute prioritisation exercise will likely differ considering the study subject and the availability of previous relevant research.

It is often difficult to predict or know in advance the number of development stages required for any particular study [9]. We propose that this design framework offers structure to researchers to apply to inform design decisions. The exploratory phase and involvement of subject experts beyond the research team is crucial to avoid narrowing the scope too early. After this stage, a structured exercise to agree priorities can be useful to understand those attributes that are strong influences or demonstrate high variability on a decision of interest. Depending of the aims of the research, one or both of these issues may prove more important to consider in DCE design. The use of the ‘think aloud’ approach is a very useful technique for pilot testing prior to data collection. It can alert researchers to ambiguous questions or terms, explore usability of the instrument and can highlight if participants feel the choice set is missing important information required to make a clear decision. The expert panel, comprising individuals with various perspectives and expertise on the decision of interest, was a useful resource during the research and we would advocate the use of such a panel to support decision-making in DCE design. It also enabled consideration of attributes that could potentially exert an unconscious influence on decision-making (as suggested by the evidence base) that may not have emerged during the qualitative research.

Among the challenges inherent in our approach is the valuation of data and how best to use the multiple data sources that were generated during the development process. Stages 1–4 shaped the study in terms of mode of administration and facilitated selection of variables identified as likely to be contributing most to variation in the decision of interest. Practical issues such as adherence to good practice guidance on design of survey questionnaires to maximise interpretability and response rates [40, 41] are also key considerations, including establishing predicted sample size informed by robust estimates of population size and response rates to similar studies. Sample size estimates are typically determined by the number of attributes and levels and what constitutes a meaningful effect size in the context of the study. These aspects are often not known a priori and therefore it can be challenging to incorporate this in the design planning process. We believe the steps described in this paper can be adopted and adapted to suit many other areas of research to inform appropriate DCE design. We encourage others to replicate the approach adopted here to explore the appropriateness of the design method to other domains.

## Conclusions

The sub-optimal reporting on crucial aspects of DCE design makes a strong case for minimum reporting guidelines. The onus is on both researchers and journal editors to ensure a minimum reporting standard is adhered to in order to indicate quality and bestow confidence in DCE results. Our five-stage design process has demonstrated that adopting a rigorous staged approach to the development of DCEs yields enhanced face validity of the choice task and strengthens the external validity of findings. The iterative, transparent development process described here could be applied to other settings and index decisions and would be a valuable adjunct to existing guidance on development of DCEs.

## Additional files

Additional file 1: On-line structured prioritisation exercise (SPE). Full survey used to collect data. (DOCX 42 kb)

Additional file 2: DCE pilot testing protocol. Interview schedule for cognitive interviewing and pilot testing of DCE. (DOCX 18 kb)

Additional file 3: Stage 3 (Phase 1): Results of on-line survey (SPE). Table presenting results of SPE online survey. (DOCX 22 kb)

Additional file 4: Operational definitions for variable attribute levels in the DCE. Table presenting definitions used for attribute levels in the DCE. (DOCX 13 kb)

## Abbreviations

COMPASS: COMPuterised decision aid for stroke thrombolysis
DCE: Discrete choice experiment
ISPOR: International Society for Pharmacoeconomics and Outcomes Research
mRS: modified rankin scale
NIHSS: National Institute of Health Stroke Scale
SPE: Structured prioritisation exercise

## References

[1] Ryan M, Gerard K (2003). Using discrete choice experiments to value health care programmes: current practice and future research reflections. Appl Health Econ Health Policy.

[2] Lancsar E, Louviere J (2008). Conducting discrete choice experiments to inform healthcare decision making. Pharmacoecon.

[3] Laver K, Rehab M, Ratcliffe J, George S, Lester L, Walker R, Burgess L, Crotty M (2011). Early rehabilitation management after stroke: what do stroke patients prefer?. J Rehabil Med.

[4] Coast J, Al-Janabi H, Sutton EJ, Horrocks SA, Vosper AJ, Swancutt DR, Flynn TN (2012). Using qualitative methods for attribute development for discrete choice experiments: issues and recommendations. Health Econ.

[5] Reed Johnson F, Lancsar E, Marshall D, Kilambi V, Mühlbacher A, Regier DA, Bresnahan BW, Kanninen B, Bridges JF (2013). Constructing experimental designs for discrete-choice experiments: report of the ISPOR conjoint analysis experimental design good research practices task force. Value Health.

[6] Bridges JF, Hauber AB, Marshall D, Lloyd A, Prosser LA, Regier DA, Johnson FR, Mauskopf J (2011). Conjoint analysis applications in health—a checklist: a report of the ISPOR good research practices for conjoint analysis task force. Value Health.

[7] Abiiro G, Leppert G, Mbera G, Robyn P, De Allegri M (2014). Developing attributes and attribute-levels for a discrete choice experiment on micro health insurance in rural Malawi. BMC Health Serv Res.

[8] Hiligsmann M, van Durme C, Geusens P, Dellaert BGC, Dirksen CD, van der Weijden T, Reginster J-Y, Boonen A (2013). Nominal group technique to select attributes for discrete choice experiments: an example for drug treatment choice in osteoporosis. Patient Preference and Adherence.

[9] Coast J, Horrocks S (2007). Developing attributes and levels for discrete choice experiments using qualitative methods. J Health Serv Res Policy.

[10] Vass C, Rigby D, Payne K (2017). The role of qualitative research methods in discrete choice experiments: a systematic review and survey of authors. Med Decis Mak.

[11] Sepucha KR, Matlock DD, Wills CE, Ropka M, Joseph-Williams N, Stacey D, Ng C, Levin C, Lally J, Borkhoff CM, Thomson R. “It’s valid and reliable” is not enough: critical appraisal of reporting of measures in trials evaluating patient decision aids. Med Decis Mak. 2014;34(5):560–6. 10.1177/0272989X14528381 PMC4190105 24713692

[12] Wardlaw JM, Murray V, Berge E, del Zoppo G, Sandercock P, Lindley RL, Cohen G (2012). Recombinant tissue plasminogen activator for acute ischaemic stroke: an updated systematic review and meta-analysis. Lancet.

[13] Sandercock P, Wardlaw JM, Lindley RI, Dennis M, Cohen G, Murray G, Innes K, Venables G, Czlonkowska A, Kobayashi A (2012). The benefits and harms of intravenous thrombolysis with recombinant tissue plasminogen activator within 6 h of acute ischaemic stroke (the third international stroke trial [IST-3]): a randomised controlled trial. Lancet.

[14] Royal College of Physicians Intercollegiate Stroke Working Party. National clinical guideline for stroke (4th EDN) Royal College of Physicians. London: The Royal College of Physicians are the publishers; 2012.

[15] Jauch EC, Saver JL, Adams HP, Bruno A, Demaerschalk BM, Khatri P, McMullan PW, Qureshi AI, Rosenfield K, Scott PA (2013). Guidelines for the early management of patients with acute ischemic stroke a guideline for healthcare professionals from the American Heart Association/American Stroke Association. Stroke.

[16] SSNAP National results summary report, based on stroke patients admitted to and/or discharged from hospital between April–June 2014 2014 [https://www.strokeaudit.org/].

[17] Murtagh MJ, Watson DLB, Jenkings KN, Lie MLS, Mackintosh JE, Ford GA, Thomson RG (2012). Situationally-sensitive knowledge translation and relational decision making in Hyperacute stroke: a qualitative study. PLoS One.

[18] Hacke W, Donnan G, Fieschi C, Kaste M, Von Kummer R, Broderick J, Brott T, Frankel M, Grotta J, Haley E (2004). Association of outcome with early stroke treatment: pooled analysis of ATLANTIS, ECASS, and NINDS rt-PA stroke trials. Lancet.

[19] Ford GA, Rodgers H, Thomson RG, al. e: Development and evaluation of hyperacute services for patients with acute Stroke In*.*: NIHR Programme Grant; 2007.

[20] Flynn D, Nesbitt DJ, Ford GA, McMeekin P, Rodgers H, Price C, Kray C, Thomson RG. Development of a computerised decision aid for thrombolysis in acute stroke care. BMC Med Inform Decis Mak. 2015;15(1):6. 10.1186/s12911-014-0127-1 PMC4326413 25889696

[21] Dirks M, Niessen LW, Koudstaal PJ, Franke CL, van Oostenbrugge RJ, Dippel DW (2007). Intravenous thrombolysis in acute ischaemic stroke: from trial exclusion criteria to clinical contraindications. An international Delphi study. J Neurol Neurosurg Psychiatry.

[22] Eissa A, Krass I, Bajorek B (2012). Barriers to the utilization of thrombolysis for acute ischaemic stroke. J Clin Pharm Ther.

[23] Flynn D (2003). Non-medical influences upon medical decision-making and referral behavior: an annotated bibliography: greenwood publishing Group.

[24] Meurer WJ, Majersik JJ, Frederiksen SM, Kade AM, Sandretto AM, Scott PA (2011). Provider perceptions of barriers to the emergency use of tPA for acute ischemic stroke: a qualitative study. BMC Emerg Med.

[25] Kwan J, Hand P, Sandercock P (2004). A systematic review of barriers to delivery of thrombolysis for acute stroke. Age Ageing.

[26] Shamy MC, Jaigobin CS (2013). The complexities of acute stroke decision-making a survey of neurologists. Neurology.

[27] SINAP—latest results: January–December 2012**.** [http://www.rcplondon.ac.uk/resources/sinap-latest-results].

[28] Pope C, Ziebland S, Mays N (2000). Analysing qualitative data. BMJ.

[29] Ryan M, Gerard K, Amaya-Amaya M. Using discrete choice experiments to value health and health care, vol. 11: Springer; 2007.

[30] BASP AUTUMN NEWSLETTER 2014**.**

[31] Van Swieten J, Koudstaal P, Visser M, Schouten H, Van Gijn J (1988). Interobserver agreement for the assessment of handicap in stroke patients. Stroke.

[32] National Institute of Health Stroke Scale [https://stroke.nih.gov/documents/NIH_Stroke_Scale.pdf].

[33] Reeves MJ, Wilkins T, Lisabeth LD, Schwamm LH (2011). Thrombolysis treatment for acute stroke: issues of efficacy and utilization in women. Women's Health.

[34] de Ridder I, Dirks M, Niessen L, Dippel D (2013). Unequal access to treatment with intravenous Alteplase for women with acute ischemic stroke. Stroke.

[35] Kent DM, Selker HP, Ruthazer R, Bluhmki E, Hacke W (2006). The stroke–thrombolytic predictive instrument a predictive instrument for intravenous thrombolysis in acute ischemic stroke. Stroke.

[36] Kent DM, Price LL, Ringleb P, Hill MD, Selker HP (2005). Sex-based differences in response to recombinant tissue plasminogen activator in acute ischemic stroke a pooled analysis of randomized clinical trials. Stroke.

[37] Willis GB (2005). Cognitive interviewing: a tool for improving questionnaire design.

[38] De Brún A, Flynn D, Joyce K, Ternent L, Price C, Rodgers H, Ford GA, Lancsar E, Rudd M, Thomson RG (2014). Understanding clinicians’ decisions to offer intravenous thrombolytic treatment to patients with acute ischaemic stroke: a protocol for a discrete choice experiment. BMJ Open.

[39] Lancsar E, Swait J (2014). Reconceptualising the external validity of discrete choice experiments. Pharmaco Economics.

[40] McColl E, Jacoby A, Thomas L, Soutter J, Bamford C, Steen N, Thomas R, Harvey E, Garratt A, Bond J. Design and use of questionnaires: a review of best practice applicable to surveys of health service staff and patients. Core Res. 2001. 10.3310/hta5310 11809125

[41] Edwards P, Roberts I, Clarke M, Diguiseppi C, Wentz R, Kwan I, Cooper R, Felix L, Pratap S (2009). Methods to increase response to postal and electronic questionnaires (review). Cochrane Database Syst Rev.

