# Supplementary material for: A novel design process for selection of attributes for inclusion in discrete choice experiments: case study exploring variation in clinical decision-making about thrombolysis in the treatment of acute ischaemic stroke
Source: BMC Health Serv Res. 2018 Jun 22;18:483. doi: 10.1186/s12913-018-3305-5 (PMC6013945; doi:10.1186/s12913-018-3305-5)
Supplement: Supplementary file 1 — On-line structured prioritisation exercise (SPE). Full survey used to collect data. (DOCX 42 kb) [file 12913_2018_3305_MOESM1_ESM.docx]

**Additional File 1.** On-line structured prioritisation exercise (SPE)

**Thrombolysis decision-making survey**

Thank you for agreeing to take part in this survey, which will be used to inform the design of a larger study (funded by the NIHR Health Services and Delivery Research Programme) to understand factors influencing clinical decision-making about thrombolysis in the treatment of acute ischaemic stroke. We are seeking the views of clinicians involved in making the final decision regarding thrombolysis for patients with acute ischaemic stroke. We anticipate that completion of this exercise will take no more than 10 minutes. Clinical decision-making about thrombolysis takes account of a range of patient-related factors and their interactions. We are seeking to understand when various patient-related factors create uncertainty in your mind as to whether to offer thrombolysis to patients with acute ischaemic stroke. Data obtained from this survey will not be individually assessed or compared with local/national guidelines or licencing criteria for thrombolysis. All responses will be strictly confidential. With your help, we are hoping to gain insight into the types of scenarios which lead to clinical variation in decision-making. Please respond to the questions below in line with your own real-world clinical decision-making. If you want to explain your response, please enter detail in the free text boxes.

Before providing a response, please carefully read each question in this survey.

Are you a clinician who is responsible for making the final decision about whether or not to offer intravenous thrombolysis to eligible acute stroke patients?

- Yes
- No

<If ‘yes’ is selected, participant may continue to the survey>

<If ‘no’ is selected, the message below is displayed and survey will skip to end>

Thank you for your interest but unfortunately you do not meet the criteria for participation in this study.

Q1. **Assuming all other indications point to thrombolysis**, at what age(s) or age range(s) would you **no** longer consider it appropriate to offer a patient presenting with acute ischaemic stroke intravenous thrombolysis? Please type your answers into the boxes below.

Highest patient age: (or enter 'none' for no upper age limit)

____________________

Lowest patient age: (or enter 'none' for no lower age limit)

____________________

If you would like to provide any further information about your responses to this question, please use the text box below:

Q2. **Assuming all other indications point to thrombolysis**, at what blood pressure levels would you **no** longer think it appropriate to offer intravenous thrombolysis? Please tick once in each list; once for systolic blood pressure (BP) and once for diastolic BP.

- Systolic BP 170-174 mm Hg
- Systolic BP 175-179 mm Hg
- Systolic BP 180-184 mm Hg
- Systolic BP 185-189 mm Hg
- Systolic BP 190-194 mm Hg
- Systolic BP 195-199 mm Hg
- Systolic BP 200-204 mm Hg
- Systolic BP 205-209 mm Hg
- No upper limit for systolic blood pressure
- Diastolic BP 95-99 mm Hg
- Diastolic BP 100-104 mm Hg
- Diastolic BP 105-109 mm Hg
- Diastolic BP 110-114 mm Hg
- Diastolic BP 115-119 mm Hg
- Diastolic BP 120-124 mm Hg
- No upper limit for diastolic blood pressure

If you would like to provide any further information about your responses to this question, please use the text box below:

Q3. Would you be willing to control a patient's high blood pressure using medication before making the final decision to administer intravenous thrombolysis?

- Yes
- No

If yes, up to what level would you be prepared to try and control a patient's blood pressure?

________________________________

Q4. Assuming all other indications point to thrombolysis, at what NIHSS scores for a patient’s current stroke would you no longer think it appropriate to offer intravenous thrombolysis? Please tick one lower and one upper value (i.e., one answer per column).

- 0
- 1
- 2
- 3
- 4
- 5
- 6
- 7
- 8
- 9
- 22
- 23
- 24
- 25
- 26
- 27
- 28
- 29
- 30
- No upper limit for NIHSS (if you believed there may some benefit to the patient)

If you would like to provide any further information about your responses to this question, please use the text box below:

Would your responses for NIHSS score(s) vary according to different patient factors? If yes, which one(s)?

______________________________________________

Q5. **Assuming all other indications point to thrombolysis**, if an acute ischaemic stroke patient is currently taking warfarin, at what international normalisation ratio (INR) level would you **no** longer think it appropriate to offer intravenous thrombolysis? Please tick the appropriate cut-off point.

- 1.4
- 1.5
- 1.6
- 1.7
- 1.8
- 1.9

If you would like to provide any further information about your responses to this question, please use the text box below:

Q6. **Assuming all other indications point to thrombolysis**, at what blood glucose (BM) level would you **no** longer think it appropriate to offer intravenous thrombolysis? Please tick as one of the following categories.

- 18.0 – 19.9 mmol/l (324 – 360 mg/dl)
- 20.0 – 21.9 mmol/l (361 – 396mg/dl)
- 22.0 – 23.9 mmol/l (397 – 431 mg/dl)
- 24.0 – 25.9 mmol/l (432 – 468 mg/dl)
- 26.0 – 27.9 mmol/l (469 – 503 mg/dl)
- No upper limit for blood glucose

If you would like to provide any further information about your responses to this question, please use the text box below:

Q7. Would you be willing to control a patient’s blood glucose level before making your final decision regarding administration of intravenous thrombolysis?

- Yes
- No

If you would like to provide any further information about your responses to this question, please use the text box below:

Q8. Assuming all other indications point to thrombolysis, please indicate at what level of social support would you no longer think it appropriate to offer intravenous thrombolysis to a patient? Please tick one or more categories as appropriate, with reference to the definition in the box below.

Social support means that an individual is cared for, has reliable assistance available from other people, and is part of a supportive social network. An individual with social support resources can access these in everyday living as well as in problem or crisis situations. This support can be emotional, tangible (e.g., financial assistance), informational and/or can offer companionship.

- Patient does not have any friends/others/family to help with everyday living or to provide emotional/informational support in times of problems/crises
- Patient has a limited supportive network (a friend or one family member) to help with everyday living or to provide emotional/informational support in times of problems/crises
- Patient has a good supportive network (friends and other people, including family) to help with everyday living or to provide emotional/informational support in times of problems/crises
- A patient’s level of social support would not influence my decision to administer thrombolysis

If you would like to provide any further information about your responses to this question, please use the text box below:

Q9. Do you take patient frailty into account when making decisions about thrombolysis for patients with acute ischaemic stroke?

- Yes
- No

<If yes, the following two additional questions will appear in survey>

Please could you describe how you assess patient frailty?

_________________________________

In what way would patient frailty influence your decision to offer thrombolysis (giving examples if possible)?

_________________________________

Q10. **Assuming all other indications point to thrombolysis**, at what level of pre-stroke cognitive status would you consider **inappropriate** to offer intravenous thrombolysis to a patient with acute ischaemic stroke? With reference to the definitions of cognitive impairment below, please tick one category.

Mild cognitive impairment: Patient experiences increased forgetfulness, slight difficulty concentrating, decreased work performance. Patient has difficulty finding the right words.

Moderate cognitive impairment: Patient experiences difficulty concentrating, decreased memory of recent events, and difficulties managing finances or traveling alone to new locations. Patient may have trouble completing complex tasks efficiently or accurately and may be in denial about their symptoms.

Severe cognitive impairment: Patient requires extensive assistance to carry out daily activities. Patient forgets names of close family members and has little memory of recent events but may remember some details of earlier life. Patient has difficulty counting down from 10, finishing tasks and the patient’s ability to speak is in decline.

- Mild cognitive impairment
- Moderate cognitive impairment
- Severe cognitive impairment
- I would not rule out thrombolysis based on a patient’s level of cognitive impairment

If you would like to provide any further information about your responses to this question, please use the text box below:

Q11. **Assuming all other indications point to thrombolysis**, what is the earliest time after a laparotomy for a perforated duodenal ulcer that you would consider it appropriate to offer thrombolysis? Please tick one category.

- 1 week
- 2 weeks
- 3 weeks
- 1 month
- 2 months
- ≥ 3 months
- No time limit would influence my decision

If you would like to provide any further information about your responses to this question, please use the text box below:

Q12. **Assuming all other indications point to thrombolysis**, how soon after Percutaneous Coronary Intervention (coronary angioplasty and stent insertion) would you consider it appropriate to offer intravenous thrombolysis? Please tick one category.

- 1 week
- 2 weeks
- 3 weeks
- 1 month
- 2 months
- ≥3 months
- No time limit would influence my decision

If you would like to provide any further information about your responses to this question, please use the text box below:

Q13. **Assuming all other indications point to thrombolysis**, how soon after a previous ischaemic stroke with reference to severity of previous stroke (assessed with NIHSS) would you consider it appropriate to offer intravenous thrombolysis? Please tick one timescale for each degree of stroke severity (mild, moderate and severe), i.e., one response per column.

|  | Previous mild stroke (NIHSS <6) | Previous moderate stroke (NIHSS 7-12) | Previous severe stroke (NIHSS ≥13) |
| --- | --- | --- | --- |
| ≤1 week |  |  |  |
| 2 weeks |  |  |  |
| 3 weeks |  |  |  |
| 1 month |  |  |  |
| 2 months |  |  |  |
| 3 months |  |  |  |
| 6 months |  |  |  |
| >1 year |  |  |  |
| Time after previous ischaemic stroke would not influence my decision to offer thrombolysis |  |  |  |
| More than 6 months but less than 1 year |  |  |  |

If you would like to provide any further information about your responses to this question, please use the text box below:

Q14. For each patient factor in the table below, please assign a rating with regard to how important you consider them to be regarding your decision-making about administration of intravenous thrombolysis for patients you have confidently diagnosed with acute ischaemic stroke.

|  | **Not important 1** | **2** | **3** | **4** | **5** | **6** | **Very important 7** |
| --- | --- | --- | --- | --- | --- | --- | --- |
| **High systolic blood pressure** |  |  |  |  |  |  |  |
| **Low systolic blood pressure** |  |  |  |  |  |  |  |
| **High diastolic blood pressure** |  |  |  |  |  |  |  |
| **Low diastolic blood pressure** |  |  |  |  |  |  |  |
| **High NIHSS score** |  |  |  |  |  |  |  |
| **Low NIHSS score** |  |  |  |  |  |  |  |
| **High blood glucose (BM)** |  |  |  |  |  |  |  |
| **Moderate/severe patient frailty** |  |  |  |  |  |  |  |
| **Anticoagulation status / INR level** |  |  |  |  |  |  |  |
| **A patient’s level of social support** |  |  |  |  |  |  |  |
| **Major surgery in past 3 months** |  |  |  |  |  |  |  |
| **Presence of diabetes at time of presentation** |  |  |  |  |  |  |  |
| **Younger age** |  |  |  |  |  |  |  |
| **Older age** |  |  |  |  |  |  |  |
| **Previous mild stroke (NIHSS < 6)** |  |  |  |  |  |  |  |
| **Previous severe stroke (NIHSS ≥ 13)** |  |  |  |  |  |  |  |
| **Pre-stroke cognitive function** |  |  |  |  |  |  |  |
| **Pre-stroke dependency status** |  |  |  |  |  |  |  |
| **Patient/relative views on thrombolysis** |  |  |  |  |  |  |  |

Many thanks for your participation in this survey that will be used to identify influential factors in clinical decision-making regarding the administration of thrombolysis for patients with acute ischaemic stroke.

**To submit your answer, please click the blue button below.**

If you have any further questions about this exercise, please contact the lead researcher using the contact details below.

[contact details here]
