# Supplementary material for: A novel design process for selection of attributes for inclusion in discrete choice experiments: case study exploring variation in clinical decision-making about thrombolysis in the treatment of acute ischaemic stroke
Source: BMC Health Serv Res. 2018 Jun 22;18:483. doi: 10.1186/s12913-018-3305-5 (PMC6013945; doi:10.1186/s12913-018-3305-5)
Supplement: Supplementary file 2 — DCE pilot testing protocol. Interview schedule for cognitive interviewing and pilot testing of DCE. (DOCX 18 kb) [file 12913_2018_3305_MOESM2_ESM.docx]

**Additional File 2.** DCE pilot testing protocol

**Understanding clinicians’ decisions to offer intravenous thrombolytic treatment to patients with acute ischaemic stroke**

**- Pilot testing protocol -**

**Draft of possible questions for semi-structured interview schedule**

**Interviewer**:

- Introduce self, background.
- Describe DCE project, its aims and expected outcomes, purpose of interview, and how long the interview should last. Assure participant they can ask for clarification on questions or choose not to answer certain questions.
- Ask permission to audio record interview. Verbally confirm ethical issues around confidentiality, anonymity and right to withdraw/end interview at any stage without reason.

**Introduction/clinician background and experience:**

- First, I’d just like to ask you about your experience and background:
  - Can you tell me about your own background? How long have you been working in your current position? (education, employment, months/years of experience and fields of experience, number of years treating acute stroke patients).
  - How long have you been offering thrombolysis where you’re based now?
- How often would you assess acute stroke patients for thrombolysis?
- Can you tell me, step by step, what is the typical assessment and decision-making process for thrombolysis that occurs for these patients?

**Decision-making process and experiences:**

- What clinical and non-clinical factors or resources do you rely upon when making decisions about whether to administer thrombolysis for acute ischaemic stroke? What are most important in decision-making?
- What issues create uncertainty for you on whether to offer thrombolysis? How do you manage this uncertainty?
- Do you incorporate patient preferences in your decisions? If yes, how?
- To what extent do licencing criteria and local or national guidelines influence your decision making?
- To what extent do your colleagues influence your decision making?
  - Do you feel comfortable approaching other clinicians for advice? How influential would their recommendation be on your decision?
- How confident are you in communicating evidence supporting treatments and the likely balance of benefit versus harm of treatment with and without thrombolysis to patients and families/carers?
- Could your recent experience with administering thrombolysis and the associated patient outcomes influence future decisions?
- As you’ve gained more clinical experience, how has this influenced your decision-making?
- Are you aware of any debates regarding the use of thrombolysis?
- What would you regard as particular ‘grey’ areas of decision-making regarding administration of thrombolysis? Can you describe a borderline/difficult case regarding the decision to administer thrombolysis which you handled?

**Perceived barriers:**

- Do you perceive any barriers to effective decision-making regarding thrombolysis? If so, could you give me examples?

**Other issues:**

- Are there other issues regarding thrombolysis that I haven’t specifically asked about or that you would like to mention or highlight as relevant to your clinical decision-making about administration of thrombolysis for stroke?

Thank you for your participation in this interview.
