# Supplementary material for: A novel design process for selection of attributes for inclusion in discrete choice experiments: case study exploring variation in clinical decision-making about thrombolysis in the treatment of acute ischaemic stroke
Source: BMC Health Serv Res. 2018 Jun 22;18:483. doi: 10.1186/s12913-018-3305-5 (PMC6013945; doi:10.1186/s12913-018-3305-5)
Supplement: Supplementary file 3 — Stage 3 (Phase 1): Results of on-line survey (SPE). Table presenting results of SPE online survey. (DOCX 22 kb) [file 12913_2018_3305_MOESM3_ESM.docx]

**Additional File 3:** Stage 3 (Phase 1): Results of on-line survey (SPE)

| **Factor** | **Potential levels (grey areas)** | **Evidence** | **Rationale for inclusion/exclusion** |
| --- | --- | --- | --- |
| **A) Systolic Blood Pressure (SBP)** | 180-184 mm/Hg  185-189 mm/Hg  190-194 mm/Hg | -Identified as upper cut off points in the SPE  -Qualitative work indicated that was SBP was a highly influential factor  - Dirks et al. ([19](#_ENREF_19)) Delphi study agreement of >185/110 as cut-off  Kent et al. ([36](#_ENREF_36)):higher SBP= less benefit from thrombolysis  -NICE (2008) guidelines state that SBP reduction to 185/110 mmHg or lower should be considered in people who are candidates for thrombolysis. | -Most important factors identified in structured prioritisation ranking exercise (SPE) and a primary issue identified in qualitative exploratory work. |
| **B) SBP control** | -SBP at decision time after attempt to reduce  - SBP at patient presentation (no attempt to reduce SBP made) | -The level at which a clinician would consider lowering SBP a separate issue to at what level they are prepared to treat  -11 out of 12 clinicians who responded to the SPE reported they would attempt to reduce SBP if necessary |  |
| **C) Diastolic blood pressure (DBP)** | 100-104 mm/Hg  105-109 mm/Hg  110-114 mm/Hg  115-119 mm/Hg | -Delphi study ([19](#_ENREF_19)) agreement of 110 as cut-off | Ranked 3^rd^/19 in SPE, indications of variation in acceptable upper level. |
| **D) NIHSS score / stroke severity** | Lower end: 0, 1, 2, 3  Upper end: >25 | -Qualitative work identified NIHSS ≤3 as creating uncertainty around offer of thrombolysis; 4-25 = safe zone; >25 = higher risk and reported as cut-off for some.  -SPE: clinicians commented that aphasia is more disabling than NIHSS suggests and this would be an important consideration (Qualitative work indicated NIHSS not always considered to be reliable indicator of severity) | -Ranking in SPE table:10/19  -Evidence for variance in acceptable lower and upper limit |
| **E) Pre-stroke cognitive status/capacity of patient** | -No cognitive impairment  -Moderate cognitive impairment  -Severe cognitive impairment | -Identified as an important factor by Shamy et al. ([24](#_ENREF_24))  -Emerged as influential in qualitative findings (to varying degrees and depending on other factors including NIHSS, dependence on others for activities of daily living, impact on quality of life)  -SPE: Severe cognitive impairment would influence decision-making of 64% of respondents; 27% reported no influence. Suggested that decision is influenced by the patient’s functional status. | -Ranked 8^th^/19 in SPE  -Little previous research on this. |
| **F) Patient’s pre-stroke dependency status (activities of daily living-ADL)** | Living independently /  24 hour support with ADL (nursing home)  Pre-stroke mRS > 2 | -Shamy et al. ([24](#_ENREF_24)): clinicians less likely to offer thrombolysis to patients who were residents of nursing homes. | -Ranked 4^th^/19 in SPE |
| **G) Frailty** | Using pre-stroke mRS (2-3)  Based on reliance on others / presence of  Comorbidities or use of composite factor / absence or degrees of frailty | -Major issue in qualitative work: clinicians concerned/nervous about treating very frail, elderly patients; concern re: falls and potential underlying injuries.  -SPE: 82% reported taking frailty into account when making thrombolysis decisions so this is clearly an important influential factor on decision-making. | -Ranked 5^th^/19 in SPE table  -Difficulty operationalising gradient of frailty; could include as dichotomous variable: frail/not frail. |
| **H) Patient age** | General agreement on no upper limit;  Lower age limit 14-18 | -Evidence indicates patients aged >80 receive similar benefit than those aged <80, particularly when treated earlier ([9](#_ENREF_9)) | -Not considered important in SPE.  -Some more likely to adhere to licence than others. |
| **I) Anticoagulation status/ INR level** | <1.6  <1.7  <1.8 | -Qualitative work found variation in acceptable levels (1.5- 1.9)  -SPE indicated grey area was between 1.6-1.8  -Recent evidence suggests thrombolysis when INR ≤ 1.7 does not increase mortality risk/risk of bleeding ([46](#_ENREF_46)); observational studies of bleeding risk among warfarin-treated patients receiving thrombolysis have been small and inconsistent ([47](#_ENREF_47)) | -Ranked 2^nd^/19 in SPE  -May be possible to include within subset of patients. |
| **J) Patient/ relative preferences re: thrombolysis** | -Patient lacking capacity and family not present (best interests)  - Relatives in favour of thrombolysis  -Relatives against thrombolysis | Qualitative work - difficulty in communication risk/benefit information; concern patients/family did not fully understand/remember info under such stress. Some clinicians expressed discomfort making decision on behalf of patients when family members not present.  -Preferences of family/patients re: thrombolysis cited as a barrier to optimal decision-making ([22](#_ENREF_22))  -Review papers ([20](#_ENREF_20), [23](#_ENREF_23)) found evidence that delays in obtaining informed consent was considered to be a factor that delayed treatment delivery. | -Ranked 6^th^/19 in SPE  -Challenging to operationalise in DCE |
| **K) Major surgery in past 3 months** | -Percutaneous Coronary Intervention (no time limit, less than 1 week)  -Laparotomy for a perforated duodenal ulcer (SPE grey areas: 2, 3, 4 weeks) | - Failure to reach agreement in Delphi exercise re: time since previous surgery ([19](#_ENREF_19)) | -Ranked 7^th^/19 in SPE  -Variation in acceptable times since major surgery |
| **L) Blood glucose level** | 22-23.9  24-25.9  26-27.9 | -SPE: variability among respondents; 25% said no upper limit  -Delphi study agreement of glucose levels 2.7-22 mmol/l ([19](#_ENREF_19)) | -Indications of variability between clinicians |
| **M) Willingness to treat to lower blood glucose level** | -Attempt has been made or has not yet been made to lower blood glucose | -SPE: 66% would not seek to control blood glucose level before making thrombolysis decision. |  |
| **N) Presence of diabetes** | -History of Type 2 diabetes  -No history of Type 2 diabetes | -Patients with diabetes had better outcomes when treated with thrombolysis than controls with diabetes ([48](#_ENREF_48)) | -Ranked 14^th^/19 in SPE |
| **O) Comorbidities** |  | Qualitative work: e.g., undergoing cancer treatment, aneurysms |  |
| **P) Previous stroke** | -Moderate stroke NIHSS 7-12 (within previous 3mths)  -Severe stroke NIHSS ≥ 13 (within previous 3 months) | -Patients with previous stroke had better outcomes when treated with thrombolysis than controls with previous stroke ([48](#_ENREF_48)) | -From SPE: Previous severe stroke ranked as more important (ranking: 10^th^) than previous mild stroke (13^th^) |
| **Q) Time since onset of symptoms** | 3~4.5 hours  >4.5 and < 5-6 hours? |  | -Qualitative work: 4.5 hours was upper cut off for most, but some clinicians more willing to accept uncertainty around onset time |
| **R) Level of social support** | -No/or minimal social support  -High level of social support | -All SPE respondents indicated that a patient’s level of social support would not impact on their decision-making about thrombolysis |  |
