# Supplementary material for: A novel design process for selection of attributes for inclusion in discrete choice experiments: case study exploring variation in clinical decision-making about thrombolysis in the treatment of acute ischaemic stroke
Source: BMC Health Serv Res. 2018 Jun 22;18:483. doi: 10.1186/s12913-018-3305-5 (PMC6013945; doi:10.1186/s12913-018-3305-5)
Supplement: Supplementary file 4 — Operational definitions for variable attribute levels in the DCE. Table presenting definitions used for attribute levels in the DCE. (DOCX 13 kb) [file 12913_2018_3305_MOESM4_ESM.docx]

**Additional File 4.** Operational definitions for variable attribute levels in the DCE

| **Attribute** | **Level** | **Definition** |
| --- | --- | --- |
| **NIHSS score**  **(stroke severity)** | NIHSS 2 (with aphasia) | Mild-moderate aphasia (+1), LOC question (+1) |
|  | NIHSS 2 (without aphasia) | Right arm (drift +1), right leg (drift +1) |
|  | NIHSS 5 (with aphasia) | Right leg (some effort against gravity: +2), Drift in right arm (+1), Mild-moderate aphasia (+1), LOC question (+1) |
|  | NIHSS 5 (without aphasia) | Right leg (some effort against gravity +2), right arm (no effort against gravity +3) |
|  | NIHSS 14 | Face (Partial paralysis +2), right arm (no movement +4), right leg (no movement +4), dysarthria (severe +2), sensory loss (severe to total +2) |
|  | NIHSS 23 | LOC questions (+2), LOC commands (+1), Best gaze (+1), Facial palsy (+2), visual fields (complete hemianopia +2), No effort against gravity in right arm (+3), No movement in right leg (+4), sensory loss (severe to total +2), aphasia (severe +2), dysarthria (severe +2), extinction/inattention (+2) |
| **Pre-stroke cognitive functioning** | Moderate dementia | Able to hold limited conversation but often requires prompting for daily routine. Forgetful of recent events and people’s names. |
|  | Severe dementia | Cannot be left unsupervised. Wanders. No meaningful conversation. Unaware of time and place. |
| **Pre-stroke dependency status (using modified Rankin scale** | mRS 1 | No significant disability. Able to carry out all usual duties and activities. |
|  | mRS 3 | Moderate disability. Requires some help, but able to walk without assistance. |
|  | mRS 4 | Moderately severe disability. Unable to walk without assistance and unable to attend to own bodily needs without assistance. |
